# Phaeohyphomycosis caused by *Cladosporium cladosporioides*: importance of molecular identification in challenging cases^☆^

**DOI:** 10.1016/j.abd.2025.501196

**Published:** 2025-08-19

**Authors:** Olivia Silva Zanetti, Maria Paula Barbieri D’Elia, Sigrid de Sousa Dos Santos, Giannina Ricci

**Affiliations:** a Department of Medicine, Universidade Federal de São Carlos, São Carlos, SP, Brazil; b Centro de Diagnóstico e Pesquisa em Biologia Molecular Dr. Ivo Ricci, São Carlos, SP, Brazil

Dear Editor,

The genus *Cladosporium* includes dematiaceous fungi that are widely distributed and frequently contaminate the environment, present in plants and soil, although some species, such as *C. cladosporioides*, *C. herbarum*, *C. oxysporum*, and *C. sphaerospermum*, are pathogenic, causing superficial and deep skin infections.^1^, ^2^

The most typical lesions present as nodules, plaques, suppurative ulcers, or crusted, ecthyma-like lesions located in areas of trauma or skin involvement.^3^, ^4^ Misdiagnoses are common due to overlapping presentations with bacterial abscesses, eczema, or other fungal infections.^1^, ^4^ This case describes a patient with a fungal infection of late etiological diagnosis, an insidious course, and a severe clinical outcome.

A 75-year-old male patient was seen in the emergency room with asthenia and skin ulcers. He reported reddish and painful nodules for eight months, mainly on his limbs, which developed into ulcers, some resolving spontaneously, with worsening over the last twenty days. He had previously been seen in an outpatient clinic, where two biopsies were performed from different sites, with inconclusive anatomopathological findings, and was treated with the hypothesis of a skin and soft tissue infection unresponsive to antibiotic therapy.

He was undergoing regular treatment for hypertension, type 2 diabetes mellitus, hyperthyroidism with goiter, and rheumatoid arthritis. He had been taking leflunomide and methotrexate for six years.

On examination, he was in fair general condition, hypoactive, and had an enlarged anterior cervical region (goiter). He had a skin lesion on his right thigh and left arm (Fig. 1), as well as dozens of painful erythematous-violaceous nodules up to 1 cm in diameter on his limbs.

**Fig. 1:**
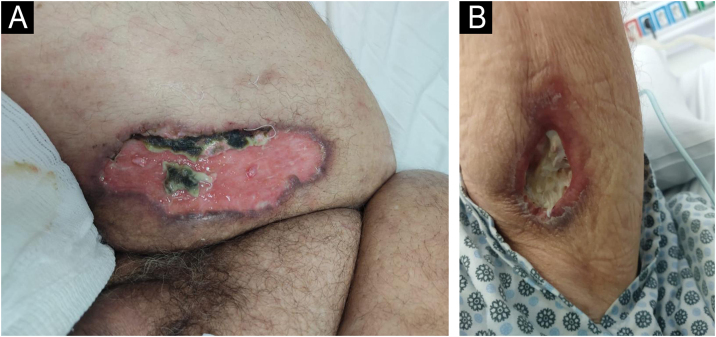
(A) Shallow ulcer in the right thigh region with necrosis of graft tissue. (B) Ulcer in the proximal lower region of the left arm.

The patient was admitted for investigation, and a biopsy was performed on a recent lesion, consistent with erythema nodosum, as the previous lesions did not show satisfactory histopathological findings. The examination revealed a perivascular lymphohistiocytic inflammatory infiltrate in the dermis and a lymphocytic infiltrate with septal neutrophils in the hypodermis, with a positive fungal test, suggesting *Histoplasma*.

Itraconazole 800 mg/day was initiated, and after thirty days, a continuous daily dose of 400 mg/day. The patient showed significant improvement in systemic symptoms and was discharged. After twelve months of regular outpatient follow-up, the patient showed complete healing of the ulcers (Fig. 2), a reduction in the number of lesions, but with recurrence of the erythema nodosum inflammation (Fig. 3). Considering a partial response to treatment, it was decided to perform a new biopsy and perform a polymerase chain reaction (PCR) for fungi and genetic sequencing of the material.

**Fig. 2:**
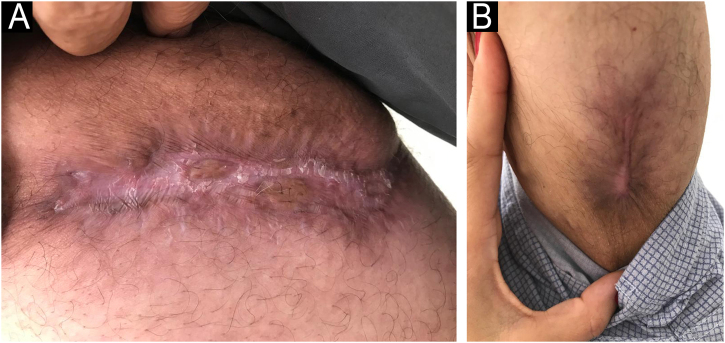
Ulcer scar on the inner right thigh after 12 months. (B) Ulcer scar on the proximal lower region of the left arm after 12 months.

**Fig. 3:**
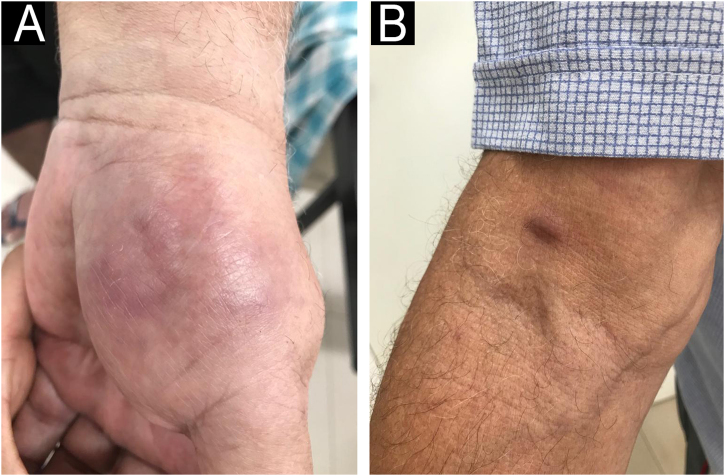
(A) Erythema nodosum in the palmar region. (B) Erythema nodosum in the cubital fossa region of the right arm.

The obtained sequence was analyzed in the BLASTn genomic database (GenBank-NCBI; https://blast.ncbi.nlm.nih.gov/Blast.cgi/) with molecular identification of the *Cladosporium cladosporioides* complex. Maximum identity or similarity was 99.7% to 100% (Fig. 4).

**Fig. 4:**
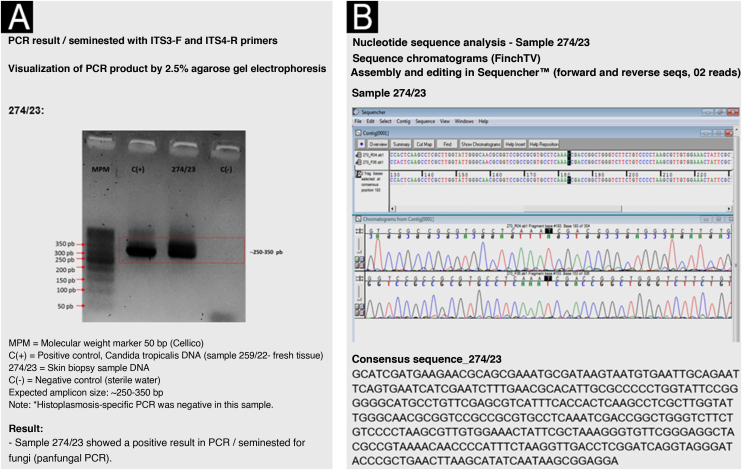
(A) PCR result for fungi. (B) Analysis of the sample's nucleotide sequences and comparison with the consensus sequence in the database.

After identifying the dematiaceous fungus, it was decided to maintain treatment with itraconazole and perform surgical resections.

While on treatment, after 24 months, the patient still had recurrent lesions. A change to amphotericin B was scheduled, but the patient was lost to follow-up. He returned to the emergency room complaining of cough and intermittent fever for three weeks. He developed severe acute respiratory failure, requiring orotracheal intubation and mechanical ventilation. A chest computed tomography scan revealed signs of atypical pneumonia, although a fungal etiology could not be ruled out. The patient progressed to septic shock and death within a few hours.

In the case described herein, the patient's age, comorbidities, and chronic medication use made him more susceptible to infection. Some cases occur in immunocompetent individuals,^3^ but severity depends on the immune status.^5^ Severe disease is more common in immunocompromised individuals (e.g., diabetes, steroid use).^6^ These fungi invade the skin through previous lesions, produce proteolytic enzymes, and resist phagocytic action, making them highly infectious to the skin, although they can rarely spread to other organs.^2^, ^3^

Itraconazole is the systemic antifungal agent of choice, administered for three to six months, with effective treatment and lesion regression in cases reported in the literature.^2^, ^3^, ^7^ Alternative treatments include voriconazole, amphotericin B, and fluconazole, the first two being used/indicated in refractory cases.^3^

The diagnosis was challenging and, as observed in this case, delayed diagnosis can lead to disease progression and unfavorable outcomes, especially with severe systemic manifestations.^4^ Molecular identification can differentiate *C. cladosporioides* from other dematiaceous fungi,^8^ and is an ally in the resolution of complex and protracted cases. Although still little used, it has potential for growth in dermatology.

## Research data availability

Does not apply.

## Scientific Editor-in-Chief

Sílvio Alencar Marques.

## Financial support

None declared.

## Authors’ contributions

Olivia Silva Zanetti: Approval of the final version of the manuscript; design and planning of the study; drafting and editing of the manuscript; critical review of the literature; critical review of the manuscript.

Maria Paula Barbieri D’Elia: Approval of the final version of the manuscript; design and planning of the study; drafting and editing of the manuscript; critical review of the literature; critical review of the manuscript.

Sigrid de Sousa Dos Santos: Approval of the final version of the manuscript; critical review of the literature; critical review of the manuscript.

Giannina Ricci: Approval of the final version of the manuscript; analysis and interpretation of the anatomopathological examination.

## Conflicts of interest

None declared.
